# An unusual cause of unilateral epistaxis: a leech in the nose

**DOI:** 10.1590/S1678-9946202567012

**Published:** 2025-02-17

**Authors:** Murat Yaşar, Fatma Atalay

**Affiliations:** 1 Kastamonu University, Faculty of Medicine, Department of Otorhinolaryngology, Kastamonu, Turkey

**Keywords:** Epistaxis, Leeches, Hirudins, Foreign bodies

## Abstract

Leeches are segmental worms commonly found in fresh water in tropical regions. They can enter the human body via the consumption of contaminated water or through the mouth and nose during washing, generally affecting the upper airway and digestive tract. During the blood-sucking process, the leech releases the anticoagulant enzyme “hirudin” from the wound site into the host’s circulation together with an anesthetic to prevent the host from feeling its attachment. Leech endoparasitism is a very rare cause of epistaxis. We report a case of a living leech lodged in the posterior nasal floor in a patient that presented to the emergency department with unilateral epistaxis and a difficult diagnosis.

## INTRODUCTION

Leeches are blood-sucking endoparasites and a very rare cause of foreign bodies in the airways. They generally inhabit fresh water. Transmission to humans generally occurs when using contaminated water for washing, swimming, or drinking. Leeches can enter the pharynx, larynx, trachea, and bronchi through the mouth and nose, which can result in potentially fatal complications^1,2^.

Epistaxis is a common symptom and an emergency ear, nose, and throat (ENT) condition. It can be either bilateral or unilateral. Frequent causes of unilateral epistaxis include septum deviation, nasal or nasopharyngeal tumors, and foreign bodies. However, leeches are responsible for only a very small portion of foreign bodies seen in the airways and are very rare as causes of unilateral epistaxis. Leeches have been reported as nasal foreign bodies in few publications^3,4^.

We report a case of a living leech lodged in the posterior nasal floor in a patient that presented to the emergency department with unilateral epistaxis and a difficult diagnosis.

## CASE REPORT

**Figure 1:**
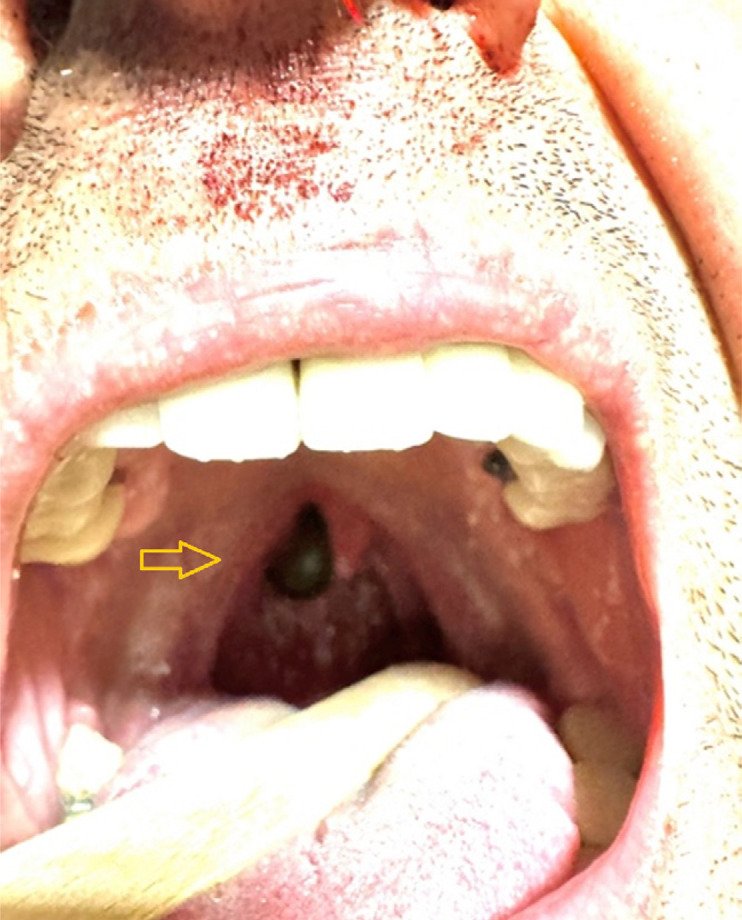
On oral examination, a green-brown mobile tissue compatible with a large live formation in the nasopharynx behind the uvula was detected.

A 41-year-old Turkish man presented to the Kastamonu Training and Research Hospital, Türkiye, due to bleeding from the left nostril that persisted for the previous two weeks. He had been examined at the ENT clinic of a local hospital the previous week at which nasal mucosa cauterization was performed on the left nasal cavity. Test carried out in the emergency department yielded negative evidence on hemorrhagic or thrombotic disorders. The patient had no history of chronic disease, nasal trauma, foreign body, fever, ecchymotic patches, dysphagia, or drug use. His hemoglobin level was 13.3 mg/dL (reference range 12–16 mg/dL), his nutrition was adequate, and no anemia findings were observed. The green-brown organism that appeared and disappeared, exhibiting twisting movements toward the oropharynx behind the uvula, was observed at oropharyngeal examination (Figure 1).

Endoscopic examination performed using a 0 degree 2.7 mm rigid telescope without anesthesia revealed that the nasal septum was deviated to the right, with mucosal ulceration associated with the cauterization present in the left Little’s region. In addition, a moving organism attached to the mucosa on the posterior floor of the left nasal cavity, close to the choanae, was detected. Under endoscopic visualization, the central part of the body of the organism was gripped using Hartmann nasal dressing forceps through oral route and withdrawn with a rapid extraction movement. It was identified as a leech.

**Figure 2:**
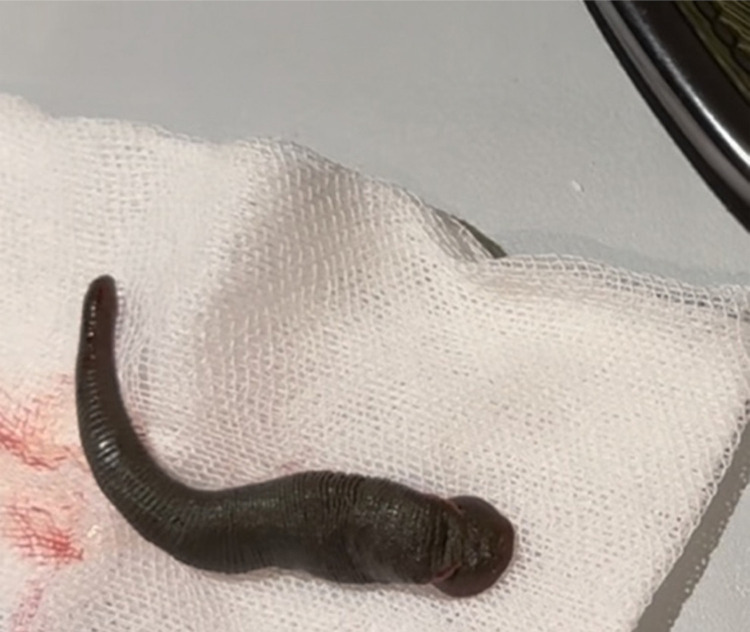
The leech was a green-brown color with a larger posterior and a smaller anterior sucker.

The leech was a green-brown color, with the posterior part being larger than the anterior (Figure 2). The bleeding stopped immediately following the extraction of the leech. Interestingly, the patient reported not consuming water from unsafe sources. He also had no history of drinking water from his hands or directly from sources without knowing its content. He reported generally drinking purified water, and occasionally going for forest walks. The patient became asymptomatic and was duly discharged from the hospital. Written informed consent was obtained from the patient who participated in this case report.

## DISCUSSION

Intranasal or nasopharyngeal foreign bodies frequently cause epistaxis. Although intranasal bodies generally result in unilateral epistaxis, cases of leeches causing epistaxis are very rare^3^. However, it is a potentially dangerous situation and requires emergency intervention.

Leeches are segmental worms that live in fresh water. *Hirudinea granulosa* and *Hirudinea viridis* are species capable of invading humans^5^. During the blood-sucking process, the leech releases the anticoagulant enzyme “hirudin” from the wound site into the host’s circulation together with an anesthetic to prevent the host from perceiving the attachment^3,6^. Leeches can progress as far as the lower respiratory system and cause severe complications by entering the body through the nose or mouth^7^. Also, pruritus, limb edema, local irritation, skin erythema, significant increase in prothrombin time (PT), international normalized ratio (INR) and erythrocyte sedimentation rate (ESR) values and decrease in hemoglobin and hematocrit levels are other complications reported with leech infestation^8^. Therefore, they should be considered in the differential diagnosis of patients with epistaxis^7^.

The most common site of leech invasion in humans is the nose, and the most marked symptom is epistaxis.^3^Endoparasitism generally persists for lengthy periods before intervention, due to the absence of pain and the lack of easily visible area involved. Physicians are therefore advised to suspect leech infestation in cases of recurrent spontaneous epistaxis of unknown etiology in tropical regions, and detailed endoscopic examination of the bilateral nasal cavities is important^9^.

A green-brown, moving foreign body in an affected nasal cavity or nasopharyngeal or oropharyngeal regions at clinical examination is suggestive of leech infestation^10^. The removal of leeches is simple. In adults, once airway safety has been established, it is sufficient to grasp the live leech by the body using an appropriate forceps and apply a rapid extraction motion^5,10^. After administering hypertonic saline solution into the nasal cavity, the leech can be paralyzed and then captured and pulled out. In addition, the application of physiological saline, vinegar, turpentine oil and alcohol can help remove the leech^11^. In this case, the middle part of the body of the leech was grasped using the Hartmann nasal dressing forceps and removed from the oropharynx without anesthesia.

In our case, the leech was close to the choana in the posterior part of the left nasal cavity. It was missed by the general practitioner since it was not visible in the anterior rhinoscopic examination and in the oropharynx due to the twisting movements of the organism. In addition, a week before the patient presented to the emergency department, the nasal mucosa in the left anterior septal regions was cauterized at a local hospital by an ENT specialist, who did not perform an endoscopic examination, due to a suspicion of septum deviation-associated epistaxis. A live leech can move about in the nasal cavity, and due to the anatomical structure of the nose, it can settle in regions that are difficult to detect even using endoscopy. It can therefore be missed by even the most experienced ENT specialist.

## CONCLUSION

In conclusion, leech endoparasitism is a very rare cause of epistaxis. Leech-related epistaxis can be refractory. A high index of suspicion of leech infestation is therefore required in cases of unresolved refractory epistaxis, especially in cases with histories suggestive of such infestation, like recently walking in the forest or drinking unclean water. Endoscopic examination must be performed in order to exclude the probability of leech invasion in the differential diagnosis of epistaxis.
